# Immunological reaction and oxidative stress after light or heavy polypropylene mesh implantation in inguinal hernioplasty: A CONSORT-prospective, randomized, clinical trial

**DOI:** 10.1097/MD.0000000000003791

**Published:** 2016-06-17

**Authors:** Marcello Donati, Giovanna Brancato, Giuseppe Grosso, Giovanni Li Volti, Giuseppina La Camera, Francesco Cardì, Francesco Basile, Angelo Donati

**Affiliations:** a Department of Surgery and Medical-Surgical Specialties, Vittorio-Emanuele University Hospital of Catania, Catania, Italy; b Department “G.F. Ingrassia,” Section of Hygiene and Public Health, University of Catania, Catania, Italy; c Department of Biomedical and Biotechnological Sciences, University of Catania, Catania, Italy; d Department of Stress Biology, Epigenetic and Biomarkers, EuroMediterranean Institute of Science and Technology, Palermo, Italy; e Department of Surgical Sciences, Organ Transplants and New Technologies, General Surgery and Week Hospital Unit, University Hospital of Catania, Catania, Italy.

**Keywords:** cytokines, immunologic reaction, inguinal hernia repair, oxidative stress, prosthetic repair

## Abstract

The relationship between mesh weight and host tissue reaction has, so far, not been fully investigated. Lightweight meshes (LWM) are thought to give less inflammatory response compared with heavyweight meshes (HWM). The present study is a randomized, controlled, double-blind clinical trial performed in 61 patients who underwent an elective inguinal hernioplasty. The primary outcome of the study was to investigate the relationship between total amount of prosthetic material (polypropylene), immunological reaction, and oxidative stress. The study was double-blinded. Sixty-one patients were recruited for the study and randomly assigned to 2 groups (groups A and B). Levels of inflammation markers (interleukin-6 [IL-6] and tumor necrosis factor-α [TNF-α]) and oxidative stress markers (reduced glutathione [GSH] and lipid hydroperoxides [LOOH]) were determined preoperatively and after undergoing inguinal hernioplasty (after 6, 72, and 288 hours), respectively, with LWM and HWM. There was no significant difference in IL-6 levels between HWM and LWM (*P* = 0.3, 0.7, 0.8 after 6, 72, and 288 hours, respectively). A statistically significant difference was found after 72 hours for TNF-α (*P* = 0.01), for GSH after 6 hours (*P* < 0.01), and after 6 and 72 hours for LOOH (*P* = 0.05, 0.01, respectively). Oxidative stress occurred at earlier time points and was pore accentuated HWM versus LWM and prodromal to TNF-α increase.

Also, in randomized clinical trial, the use of LWM gives advantages in terms of less inflammatory response when compared with HWM. Moreover, there is a significant higher oxidative stress after implantation of HWM. The intensity of oxidative stress seems to be strongly related to the amount of implanted polypropylene. (Trial registration number: NCT01090284).

## 1 Introduction

The notable development and diffusion of prosthetic surgery of the abdominal wall over the last few years has led to the introduction of lightweight meshes (LWM).^[1]^ The efficacy of inguinal hernia repair with lightweight prostheses, as well as the better or worse biotolerability with respect to those of lightweight, remains questionable in literature,^[2]^ where a clear answer still remains to be given.^[3,4]^ If there exists a connection between the quantity of material implanted, the immunological reaction to the mesh,^[5]^ the induced oxidative stress and the degree of cicatrization,^[6–8]^ and consequently the long-term result of the efficacy of the operation remains to be demonstrated,^[9]^ and is still a matter of debate.^[10]^ The impressive development of prosthetic hernia repair over the last 20 years has led industry and surgeons to research on new kinds of meshes. In the last 6 to 7 years, LWM have been introduced in clinical practice on the assumption that a lightweight prosthesis could reduce local complications, such as discomfort and chronic pain, or inguinal impairment, that had been referred to heavyweight meshes (HWM).^[4,11,12]^ However, published studies have succeeded in demonstrating a lower incidence of discomfort and pain using LWM, but some studies have indicated a higher incidence of hernia recurrence^[9,13]^; despite these results, some controversies^[27]^ still remain and the general interest for LWM has remained.^[14,15]^

Immunological response seems to play a major role in the complex mechanisms of repair following mesh implantation. To this regard, the polypropylene prostheses, which are more frequently used, induce a rapid and useful acute inflammatory response,^[16]^ followed by an incorporation of them into the area of implant, with a limited fibroblastic response ^[17]^ and a strong scar tissue.^[18]^ The inflammatory response seems to be characterized by increased levels of interleukin (IL)-6 and C-reactive protein^[6,16]^ associated with other modifications of inflammatory serum markers (fibrinogen, alpha-1 antitrypsin). The immunologic reaction to polypropylene was already studied in previous published reports. However, in the present study we studied different cytokines such as IL-6 and tumor necrosis factor-α (TNF-α) compared with the previously studied types (IL-1, interferon-γ, IL-10, fibroblast growth factor, and vascular endothelial growth factor)^[5,6,10,19,20]^ and how these variables are related to oxidative stress. IL-6 and TNF-α were separately studied in different groups and in a smaller cohort of patients, comparing traditional surgery with prosthetic repair for inguinal hernia. The main conclusion of these studies was that polypropylene mesh induces an inflammatory response that was quantitatively relevant with respect to traditional techniques. In some other reports, Di Vita et al stated that such an immunologic response was proportional to the quantity of polypropylene inserted and additionally postulated that such an increase of cytokines (IL-6, TNF-α, IL-1, etc) could be correlated to the incidence of local complications due to meshes.^[28]^ IL-6 is often induced together with TNF-α in many inflammatory conditions. However, whether IL-6 plays a pro- or anti-inflammatory role in local inflammation is not clear. Although it is commonly believed that IL-6 acts as an inducer of inflammatory genes, a recent report about IL-6 (−/−) mice indicates a crucial anti-inflammatory role by controlling the level of proinflammatory cytokines.

There are few studies on the immunological reaction to polypropylene meshes,^[11,14,15]^ few on the comparison between lightweight and heavyweight,^[19,21]^ and to the best of our knowledge, none on the oxidative stress induced by the mesh. Moreover, only one study has been published that clearly correlates the immunological reaction to the amount of prosthetic material.^[15]^ Whether the immunologic reaction depends on pore size and texture instead of materials remains questionable and controversial data have been published in the literature.^[19,22]^

The aim of this research was to evaluate a possible relationship between the amount of implanted polypropylene and immunological reactions as well as postoperative oxidative stress, and thus to evaluate, if present, the differences in the biological reaction and biotolerability between LWM and HWM on a statistically significant number of patients with a randomized prospective clinical trial.

## 2 Materials and methods

### 2.1 Patients

Between March 2010 and December 2011, 64 patients were prospectively recruited and randomized in a double blind manner for the present study. Three patients (dropout rate 4.68%) were excluded from the study (lost to follow-up, postoperative discovery of multiple sclerosis, and a previous mesh implant). The recruitment of patients took place following the random order in which they were referred to the hernia service of the General Surgery and Week Surgery Unit of the University Hospital of Catania which is a large, high complexity university hospital. After physical examination confirming the diagnosis of inguinal hernia and once surgery had been indicated, informed consent was obtained from the patient by means of a standardized form. Hernia type was registered for each patient according to Rutkow classification.^[23]^ Regarding inclusion and exclusion criteria for the study, all patients affected by primary inguinal hernia between 18 and 90 years, not previously operated with implantation of a prosthetic mesh, were enrolled. Patients affected by diabetes, cirrhosis, any chronic inflammatory disease or under corticosteroids, and/or immunosuppressive therapies (neoplastic patients) were excluded from the study. A total of 61 patients were recruited. Two patients were, after being enrolled, excluded due to the discovery of previous operations with mesh implants and an anamnestic positivity for immunological disorders (multiple sclerosis), one patient did not return after the operation to complete the requested protocol of blood analyses. These 3 patients had previously been randomly assigned to LWM. Therefore, in the end 29 patients were enrolled in LWM, whereas there were 32 in HWM. This study was carried out in accordance with the Declaration of Helsinki (2000) of the World Medical Association. The patients were not informed as to which prosthesis was implanted. The ethics committee of Policlinico-Vittorio Emanuele University Hospital of Catania approved the study, informed consent form, and the workflow of the research on March 2010, study code CH001GENI. Then the study was registered at http://clinicaltrials.gov/show/NCT01090284 (registration number assigned NCT01090284). The study ended in December 2011.

### 2.2 Study design

Patients were randomly assigned to 2 groups (random criterion: i.e., patient 1 (LWM), patient 2 (HWM): in LWM inguinal hernioplasty surgery was carried out with the use of the so-called “lightweight” type (40 g/m^2^ of polypropylene); for HWM, on the contrary, the mesh was of the “heavyweight” type (220 g/m^2^). Simple randomization was used by flipping a coin.^[24]^

The laboratory assessments were determined by another investigator without any information about sample origin (if from an LWH or HWM patient, this investigator was also blind to the randomization criteria), as well as kind of implanted mesh. Although anamnestic data of patients were collected by surgeons, the statistical evaluation was carried out by another investigator.

### 2.3 Study intervention

The 2 types of meshes had the same pore size, the same texture (monofilament polypropylene meshes), and came from the same manufacturer (HERTRA, Herniameshs.r.l. Chivasso, Turin, Italy). They differed only in weight (g/m^2^) (content of polypropylene). They did not differ for stiffness or size. For each patient, a preoperative blood test was carried out to determine the basal levels of IL-6, TNF-α, reduced glutathione (GSH), and lipid hydroperoxides (LOOH). All these components were determined on blood samples, respectively preoperatively, 6 hours after the operation, on 3rd and 12th postoperative days. All samples were frozen for reference. No perioperative preparation (including medications) was performed in all patients as for our guidelines. All the patients underwent an open local anesthesia (solution of mepivacaine 2%, 30 mL) prosthetic inguinal hernia repair as gold standard technique,^[25]^ a published variant of Trabucco's repair with the apposition of one or more plugs. Mesh fixation was performed by suturing mesh flaps around spermatic cord through 2/0 polypropylene (Prolene) stitches (Ethicon, Somerville, NJ). Wound size was about 10 cm and was closed by intradermic reabsorbible suture with 3/0 polyglactin 910 (Vicryl) (Ethicon). All surgical procedures were performed by the same experienced surgeon (surgical experience >40 years). All patients received postoperatively, ceftriaxone (1 g/die for 4 days), tramadol (1 vial), when requested by the patient to control postoperative pain. Patients were monitored in an ambulatory manner for 12 days and complications were recorded.

### 2.4 Study endpoints

The primary endpoint of the present research was to evaluate a possible relationship between the amount of implanted polypropylene and immunological reactions as well as postoperative oxidative stress, and thus to evaluate, if present, the differences in the biological reaction and biotolerability between LWM and HWM on a statistically significant number of patients with a randomized prospective clinical trial.

### 2.5 Sample dimension

According to previous studies,^[19–21,26–28]^ comparison of the means and standard deviations of TNF-α at baseline and 72 hours between the 2 intervention arms was taken into account for the calculation of the sample size. Power analysis showed that 30 participants in each arm of the trial were adequate to evaluate 2-sided standardized differences between subgroups of the study and that the investigated parameters >0.5 achieved statistical power >0.80 at 5% probability level (*P* value).

### 2.6 IL-6 and TNF-α determination

Cytokines were measured by commercially available enzyme-linked immunosorbent assay kits (R&D Systems, Minneapolis, MN). The assay was performed in accordance with the protocol provided by the manufacturer and as previously described.^[29,30]^ Briefly, sample was incubated with anti-IL-6 or anti-TNF-α, anti-rabbit immunoglobulin G, and horseradish peroxidase conjugates, in successive order. Absorbance at 450 nmol/L was measured, and cytokine concentrations were calculated from a standard curve generated with purified IL-6 or TNF-α. Each measurement was performed in triplicate and averages were reported.

### 2.7 Determination of GSH levels

Blood levels of total thiol groups (GSH) were measured in 200 μL of blood samples using a spectrophotometric assay based on the reaction of thiol groups with 2,2-dithio-bis-nitrobenzoic acid at λ=412 nm. The limit of detection for this assay is approximately 15 nmol/L.^[29,31]^ The intra-assay coefficient of variation is 4%, whereas the interassay coefficient of variation is 5.6%.

### 2.8 Determination of LOOH levels

LOOH levels were evaluated following the oxidation of Fe^2+^ to Fe^3+^ in the presence of xylenol orange at λ=560 nm. The assay mixture contained 100 mmol/L xylenol orange, 250 mmol/L ammonium ferrous sulfate, 90% methanol, 4 mmol/L butylated hydroxytoluene, and 25 mmol/L H_2_SO_4_. After a 30-minute incubation at room temperature, the absorbance was measured using a U2000 Hitachi spectrophotometer (Hitachi, Tokyo, Japan). Calibration was obtained using hydrogen peroxide (0.2–20 mmol/L).^[32,33]^ The limit of detection for this assay is approximately 0.25 nmol/L.

### 2.9 Statistical analysis

Baseline characteristics are presented as mean and standard deviation. Assumption of normal distribution of cytokines and oxidative stress markers was tested by Shapiro-Wilk test and parametric tests were used. Statistical differences between groups (light vs heavy) at different time after operation (postoperative time, 6 hours, 72 hours, 12 days) were tested by the *t* test and ANOVA (with Bonferroni correction for multiple comparisons), as appropriate. Univariate linear regression analyses were performed to identify independent predictors of serum inflammatory marker levels as continuous dependent variables. In these models, we selected as independent variables such as age, body mass index (BMI), operative time, mesh type, and number of plugs. When more than one variable was found significant at univariate analysis (probability threshold, *P* ≤ 0.05), multivariate analysis was performed to assess which was independently associated with serum inflammatory marker levels. All statistical tests were 2-tailed and a *P* ≤ 0.05 was considered significant. Data were entered into Microsoft Excel for Windows (Microsoft Corporation, Redmond, WA). Statistical analysis was performed using SPSS for Windows release 17.0 (SPSS Inc, Chicago, IL).

## 3 Results

### 3.1 Patients

No statistically significant difference was found between groups related to age and BMI (Table 1). Hernia-type distribution in our study population is shown in Table 2. No postoperative complications, such as seroma, infection, wound dehiscence, mesh infections, fistulization, or allergic reaction to the prostethic material, were observed. A difference was found regarding operative time and implanted plug number in favor of HWM (respectively 96.4 ± 21.4 vs 117.5 ± 38.6 minutes and 1.3 ± 0.6 vs 1.8 ± 1; *P* = 0.013 and *P* < 0.034) (see Table 1).

**Table 1:**
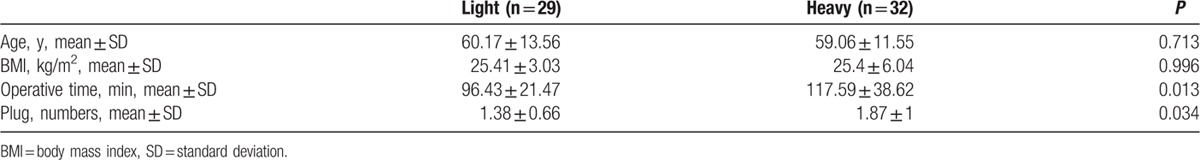
Clinical characteristics of the study population according to mesh type.

**Table 2:**
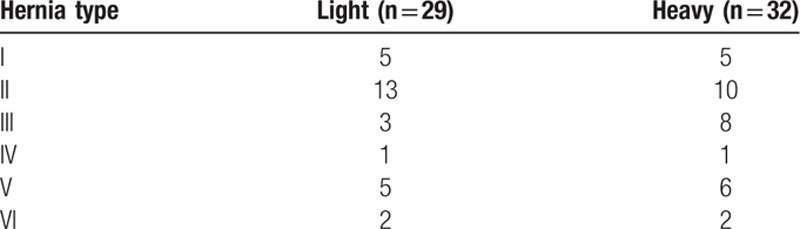
Hernia-type distribution in the studied population according to Rutkow classification.

### 3.2 Evaluation of study endpoints

**Figure 1:**
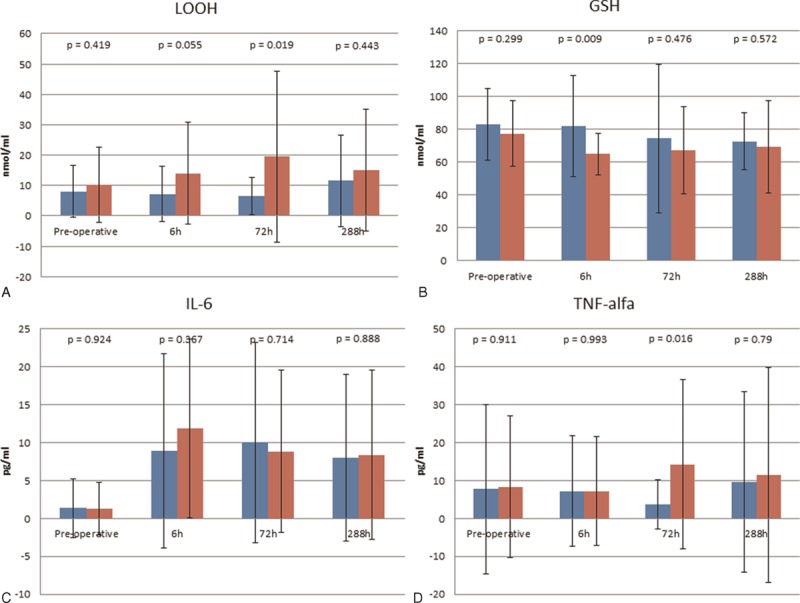
Overview of data, in blue lightweight mesh (LWM) mean values, and in red heavyweight meshes (HWM). Error bars represent standard error of the mean.

No statistical significance was found analyzing IL-6 levels, even if a trend of increase following amount of polypropylene could be found in postoperative time at 6 and 288 hours after mesh insertion (respectively preoperative, after 6, 72, and 288 hours: LWM 1.38, 8.92, 10.03, 7.99 pg/mL vs HWM 1.29, 11.87, 8.86, 8.4 pg/mL). This trend was also confirmed by TNF-α data even if gaining significance only after 72 hours (3.67 vs 14.3 pg/mL, *P* = 0.016) (Fig. 1).

Univariate linear regression analysis confirmed that TNF-α on the third postoperative day showed a tendency to increase with BMI (*P* = 0.06∗); there was also a statistical significance related to operative time (*P* = 0.013), type of mesh (more on HWM; *P* = 0.019), and number of plugs (*P* = 0.001) (see Table 3). Adjusted linear regression strongly confirmed the significance relating to plug numbers (*P* = 0.048) (see Table 4). Regarding oxidative stress, the GSH values were influenced by the kind of mesh, gaining significance (after 6 hours LWM 81.93 vs HWM 64.93 nmol/mL, *P* = 0.01). On univariate linear regression, this tendency (also if without significance) was confirmed depending mainly on age (*P* = 0.08) and mesh type (*P* = 0.06∗). Also, for LOOH we observed a higher increase after 6 and 72 hours for HWM (respectively LWM 7.16 vs HWM 13.98 nmol/mL, *P* = 0.055, and 6.61 vs 19.45 nmol/mL, *P* = 0.019). On univariate analysis, the factor mainly responsible for this difference, also this time, was mesh type (*P* = 0.02) (Table 3).

**Table 3:**
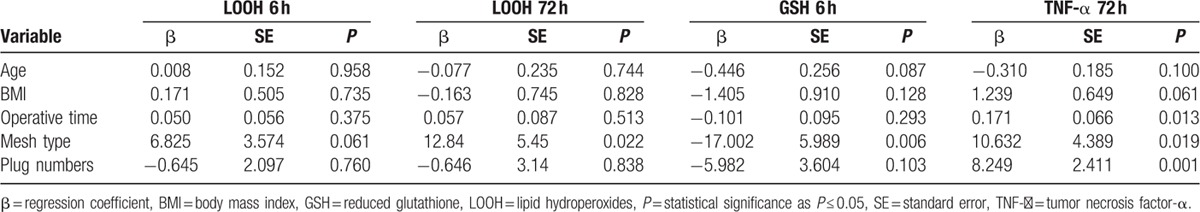
Univariate linear regression of factors associated with inflammatory markers.

**Table 4:**
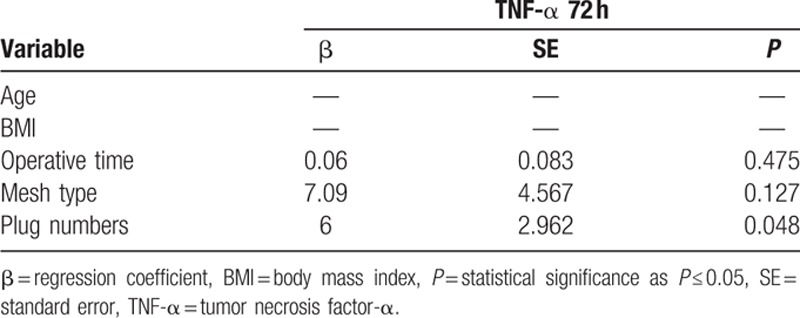
Adjusted linear regression model of factors associated with TNF-α at 72 hours.

## 4 Discussion

Our data show a correlation between number of plugs (polypropylene content) and immunologic reaction related to TNF-α on the third postoperative day. Our data also demonstrate an increase of immunologic reaction proportional to the prolongation of operative times and patient body weight and significantly related to mesh weight (more weight, more inflammation) and even number of plugs (total amount of polypropylene). A previous work by Di Vita^[26]^ showed a similar tendency but with the bias of a different contact area with the human body.^[34]^ In fact, in the above-mentioned study the authors compared a group undergoing inguinal hernia repair with a group undergoing incisional hernia repair. Apart from the bias of comparing 2 different diseases with different cicatrization capabilities of patients, in that study the authors compared 2 different amounts of prosthesis implantation but with 2 different contact areas.^[26]^ In our study these 2 biases were eliminated. Furthermore, our study was a double blind (neither the patient, nor the investigators involved in collection and analysis of data were informed about which treatment the patient received) prospective randomized clinical trial. Our work confirmed some previously published data, showing that reaction to polypropylene correlates positively not only with contact area (number of plugs) but also with overall amount of implanted material (mesh type). Regarding the statistically significant difference in our cohort it was found only on the third postoperative day for TNF, but this was mostly due to the size of our cohort of patients mainly conditioned by costs of reactants for immunological determinations. Univariate analysis confirmed this tendency giving significance for number of plugs while losing it for BMI. Taking together these data can affirm that also in a prospective clinical trial the intensity of immunologic reaction such as oxidative stress seems to be positively related to increase of overall polypropylene amount and not only with the extension of the contact area.^[34]^ As far as we know this is the only study investigating immunologic reaction on 60 patients, testifying to the difficulties of conducting such a study on large cohorts of patients. In addition, our recruitment was prospective and unlike other studies, all selection biases were preventively eliminated. On the contrary, as regards oxidative stress, the study succeeded in finding such a correlation between increase of mesh weight and oxidative stress, indirectly measured by means of antioxidant system consumption (GSH decreases and LOOH increases). The clinical significance of these data is still unclear; it remains to be elucidated whether our results are related to a higher risk of development of local complications^[35]^ and modification of meshes.^[36]^ As regards clinical practice, LWM could be considered less invasive (possibly inducing less immunologic reactions and oxidative stress) compared with HWM, even if this advantage can mean a high incidence of recurrences. Both oxidative stress and immunologic reaction seem to be linked in experimental models.^[32]^ Moreover, we could postulate that to reduce oxidative stress induced by polypropylene, L-carnitine^[37]^ could be added to the mesh to reduce oxidative effects, even if clinical experiences are needed. On the contrary, the stronger immunologic reaction (IL-6, TNF-α) due to polypropylene implantation, following some clinical studies attributing an antiseptic role to the increase of such cytokines,^[3]^ could explain the rare occurrence of polypropylene infections. Results of this study should be considered in light of the limitation that we were not able to collect data of 3 patients, and this may have led to selection bias. Moreover, the limited number of patients included in the trial may have weakened some results (i.e., LOOH at 6-hour follow-up).

To the best of our knowledge this is the first trial investigating the correlation between polypropylene meshes for inguinal hernia repair and oxidative stress, considering the interest for oxidative stress^[38,39]^ and antioxidative systems^[40,41]^ in current research. Taking into account the supposed role of oxidative stress on the evolution of local complications, we hope that our study will stimulate a new research field on the evolution of prosthetic materials in abdominal wall surgery, maybe stimulating industries to produce not only “less inflammatory” but also “less oxidative” meshes. Our study does not take into due account the genetic variability of enrolled patients. In fact, it is known that polymorphisms in IL-6 gene may results in a different inflammatory response under certain stressful condition. However, the blinded randomization may in part mitigate such bias. Finally, our sample size was sufficient to find the differences between groups of interest, but an increased number of subjects enrolled in the study may have strongly emphasized also a linear association (i.e., for both LOOH at 6 hours and TNF-α at 72 hours).

In conclusion, the immunologic reaction to polypropylene after inguinal hernioplasty is directly related not only to the contact area with the host but mainly to the overall amount of material per cm^2^. The oxidative stress is strongly related to the quantity of polypropylene for inguinal hernia repair, and is prodromal to TNF-α increase. HWM implantation causes a stronger immunologic reaction and oxidative stress in the host compared with LWM. In particular, our data suggest that LOOH and GSH are influenced by the mesh type, whereas TNF-α level is depending on the plug numbers.

As regards the clinical significance of such data we can, at the moment, only speculate; this aspect should be further investigated maybe with studies on larger cohorts of patients.
